# Predicting CD4 T‐Cell Reconstitution Following Pediatric Hematopoietic Stem Cell Transplantation

**DOI:** 10.1002/cpt.621

**Published:** 2017-05-26

**Authors:** RL Hoare, P Veys, N Klein, R Callard, JF Standing

**Affiliations:** ^1^ Centre for Mathematics and Physics in the Life Sciences and Experimental Biology University College London London United Kingdom; ^2^ Great Ormond Street Institute of Child Health University College London London United Kingdom; ^3^ Great Ormond Street Hospital for Children NHS Trust London United Kingdom

## Abstract

Hematopoietic stem cell transplantation (HSCT) is an increasingly common treatment for children with a range of hematological disorders. Conditioning with cytotoxic chemotherapy and total body irradiation leaves patients severely immunocompromised. T‐cell reconstitution can take several years due to delayed restoration of thymic output. Understanding T‐cell reconstitution in children is complicated by normal immune system maturation, heterogeneous diagnoses, and sparse uneven sampling due to the long time spans involved. We describe here a mechanistic mathematical model for CD4 T‐cell immune reconstitution following pediatric transplantation. Including relevant biology and using mixed‐effects modeling allowed the factors affecting reconstitution to be identified. Bayesian predictions for the long‐term reconstitution trajectories of individual children were then obtained using early post‐transplant data. The model was developed using data from 288 children; its predictive ability validated on data from a further 75 children, with long‐term reconstitution predicted accurately in 81% of the patients.

**Study Highlights**

**WHAT IS THE CURRENT KNOWLEDGE ON THE TOPIC?**

☑ The rate and extent of CD4 T‐cell recovery post‐stem cell transplantation has been studied separately in selected groups of patients through summarizing counts at certain time points or the time to reach a certain count.

**WHAT QUESTION DID THIS STUDY ADDRESS?**

☑ Using a mechanistic nonlinear mixed effects model, can we simultaneously model rate and extent using data from all available patients with heterogeneous diagnoses, stem cell sources, and therapeutic conditioning protocols? What are the key patient factors associated with CD4 T‐cell recovery?

**WHAT THIS STUDY ADDS TO OUR KNOWLEDGE**

☑ A single mechanistic model can be used to fit heterogeneous data on CD4 T‐cell recovery in children. The important factors associated with CD4 T‐cell reconstitution have been identified and quantified with time.

**HOW THIS MIGHT CHANGE CLINICAL PHARMACOLOGY OR TRANSLATIONAL SCIENCE**

☑ There are two major uses of the model. First, in predicting CD4 T‐cell recovery, it can be used to inform future study and/or clinical protocol design of novel conditioning protocols, and second, as a Bayesian tool to predict CD4 T‐cell recovery in individual patients to inform clinical practice.

Hematopoietic stem cell transplantation (HSCT) is used to treat a range of malignant and nonmalignant disorders, including leukemias, immunodeficiencies, metabolic disorders, hemoglobinopathies, and marrow failure. Patients who undergo HSCT usually receive conditioning to eradicate disease and reduce or ablate the host immune system to prevent rejection. This comes in the form of radiotherapy, cytotoxic chemotherapy, and antilymphocyte antibodies. Conditioning leaves patients severely immunocompromised and liable to both opportunistic infections and re‐emergence of latent infections, such as adenovirus, cytomegalovirus, and Epstein‐Barr virus. Infection constitutes a major cause of mortality from HSCT.

After HSCT, the reconstitution of some hematopoietic cells (e.g., neutrophils) is fast, taking a matter of weeks. However, the reconstitution of others, including CD4 T lymphocytes, is slow, taking months to years, requiring extended patient follow‐up post‐HSCT. CD4 T cells are crucial to immune function, and a recent study in children receiving antithymocyte globulin showed successful CD4 T‐cell reconstitution was associated with improved survival.^1^

Identifying patient characteristics associated with slow reconstitution and predicting individual reconstitution trajectories will prove useful in both designing new studies of conditioning protocols and in clinical management post‐HSCT. Predicting T‐cell reconstitution in children is complex, however, because the time scales of reconstitution are similar to that of immune system development.^2^ Furthermore, children receive HSCT for a variety of reasons and at different ages, so collating large datasets will result in heterogeneity in patient characteristics.

To date, studies have tended to use small homogeneous groups of patients and assessed reconstitution by either taking the concentration of lymphocyte subsets at certain predetermined time points after HSCT,^3^, ^4^, ^5^, ^6^ or measuring the time taken to reach predetermined concentrations.^1^, ^7^, ^8^, ^9^ These approaches do not study the entire population receiving HSCT, and through summarizing available data, only evaluate the rate or extent of the reconstitution, not both.

A mathematical model of all available data can give both the rate and extent of reconstitution by deriving a trajectory for CD4 concentration with time. Mixed‐effects modeling make it possible to fit mathematical models to the sparse, uneven, and heterogeneous data available, while removing bias by accounting for correlations in subjects' data through parameter‐level interindividual variability.^10^ Such models can be used for the design and analysis of clinical trials. Recently, it has been shown that using a mechanistic model fitted to all data points, rather than comparative statistical test at a single time point, can increase the power to detect drug effects by up to 10‐fold.^11^ Future clinical trials on new agents for conditioning using mechanistically modeled CD4 response as an outcome could, therefore, be conducted with substantially fewer patients than a traditional study design.

In this article, we present a novel mechanistic mathematical model for CD4 T‐cell reconstitution after pediatric HSCT. To delineate age‐related effects from other important covariates, we used *a priori* scaling of production and loss terms in the model. This was based on previous models taking T‐cell receptor excision circle (TREC) and Ki67 expression to infer changes in thymic output, proliferation, and loss with age.^12^, ^13^ We first used the model to identify the factors significantly associated with reconstitution, and, second, to make individualized predictions for long‐term reconstitution using these covariates and CD4 T‐cell counts from the first 6 months post‐HSCT.

## RESULTS

### Mechanistic model building

**Figure 1:**
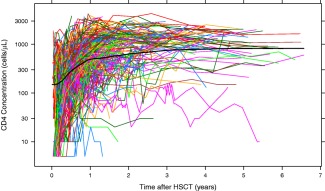
Data for CD4 T‐cell reconstitution after pediatric hematopoietic stem cell transplantation (HSCT; *n* = 319). These data were used in the model development and covariate analysis. Each colored line is the data for an individual transplant. The thick black line gives a local regression curve for the data.

**Figure 2:**
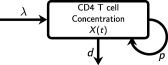
Schematic of the model. The compartment *X*(*t*) represents CD4 T‐cell concentration in the peripheral blood with time *t* after hematopoietic stem cell transplantation (HSCT). New cells output by the thymus enter the compartment at zero‐order rate λ and cells proliferate into two cells or die at first‐order rates *p* and *d*, respectively. Scaling for age was added to λ, *p*, and *d* and a function causing a time delay in the recovery of λ after transplant was used.

The raw data (CD4 T‐cell concentrations in blood) from children after HSCT are given in **Figure** 1. A one‐compartment turnover model was used whereby new cells enter the compartment from the thymus, and cells may then proliferate or die (**Figure** 2). Functions were included to account for the underlying biology of the system. The homeostatic mechanisms, and, in particular, competition for resources, such as cytokines and self‐peptide major histocompatibility complex, were represented by dependence of both proliferation and loss on cell concentrations.^14^, ^15^, ^16^, ^17^ Age dependence of proliferation and loss were included in the model to account for the dynamics of the system known to slow with age.^12^, ^13^, ^18^ Thymic output was also modeled as age dependent as the thymus involutes with age and T‐cell production decreases. Finally, the model accounts for the delay to production of T cells by the thymus after HSCT shown by analysis of previous data for TRECs and recent thymic emigrants.^3^, ^19^, ^20^ The mathematical functions used are described in the Methods section.

Parameter estimates for the model‐building dataset are given in **Table** 1. The typical CD4 T‐cell concentration returned to 90% of the expected value for age, and then followed the expected trajectory of a healthy child. On average, it took 22 months for a typical child of median age at the time of HSCT to reconstitute to this CD4 concentration for age, varying from 17 months for a 1‐year‐old to 33 months for a 10‐year‐old. The mean delay for thymic output to recover to 50% production was found to be 5 months. Thymic output recovery was fast, with the time taken to recover from 10–90% being 3.5 months. After the thymic output recovered, the thymic output for a typical child of median age was found to peak at 200 cells/day, in the region expected for a healthy child of that age.^12^, ^21^ The covariates tested are listed in **Table** 2.

**Table 1:** Typical model parameter estimates with SDs, and random effect variances with SDs

Structural model					
Parameter		Estimate	SD	Ω	SD
λ_0_	Proportion of theoretical thymic output^13^ (cells/day)	0.216	0.0711	1.57	0.55
*d*_0_	Proportion of expected loss (/day)	0.477	0.0959	1.62	0.386
*p*_0_	Proportion of expected proliferation (/day)	0.207	0.0239	0.251	0.0960
*X*_0_	Initial concentration of T cells (cells/μL)	168	21.5	1.31	0.206
λ_*h*_	Time to recovery in thymic output (days)	133	20.3	1.27	0.247
λ_*r*_	Rate of recovery in thymic output	9.66	1.36	1.22	0.431
σ	Variance of the residual error	0.219	0.0167	—	—

Covariate model				
Parameter	Covariate	Effect size	SD	*P* value
*X*_0_	Alemtuzumab	‐0.842	0.029	<0.001
*X*_0_	ATG	‐0.939	0.052	<0.001
*X*_0_	Acute GvHD	0.283	0.196	<0.001
λ_0_	Leukemia	1.32	0.442	<0.001
*p*_0_	No conditioning	‐0.844	0.025	<0.001

ATG, antithymocyte globulin; GvHD, graft‐vs.‐host disease.

Parameter estimates and the random effect variances (Ωs) were estimated from the model‐building dataset. The SDs for both the parameter means and for the variances of the random effects were found through 200 bootstrap samples using PsN version 3.5.3.^44^ The significant categorical covariates were included through multiplication of the parameter by (1+*Effectsize*), testing the null hypothesis that the effect size is zero.

**Table 2:** Percentage breakdown of the demographics and the drugs used for the patients in the datasets, all of which were tested as covariates

	M	V		M	V		M	V
	%	%	Diagnosis	%	%	HSCT	%	%
Age at HSCT, years								
0→1	16	19	Immunodeficiencies	43	40	1st	85	88
1→2	21	16	SCID	26	24	2nd	13	11
2→5	23	21	Wiskott‐Aldrich	4	7	3rd	1	1
5→10	24	31	CGD	4	8	GvHD		
10→	16	13	Leukemia	30	23	Reported	32	60
Sex			ALL	14	11	I	12	33
Male	37	32	AML	11	11	II	12	20
Female	63	68	HLH	11	7	III	6	5
Stem cells			Anemia	7	0	IV	2	1
Bone marrow	47	36	Autoimmune	3	0	Conditioning		
Peripheral blood	38	37	Lymphomas	2	0	Fludarabine	21	73
Cord blood	15	27	Viruses			Cyclophosphamide	44	16
Combinations	1	0	Cytomegalovirus			Melphalan	30	23
Donor type			Positive	32	16	Busulphan	24	41
Matched	63	52	Negative	67	81	Treosulphan	21	24
Sibling	27	19	Unknown	1	3	Alemtuzumab	50	40
Family	5	7	Epstein‐Barr virus			ATG	3	16
Unrelated	31	27	Positive	26	16	Anti‐CD45	4	3
Mismatched	32	37	Negative	38	64	Total body irradiation	14	8
Sibling	1	0	Unknown	37	3	None	13	5
Family	2	1	Adenovirus			Prophylaxis		
Unrelated	29	36	Positive	33	–	Cyclosporine	88	88
Haploidentical	4	3	Negative	67	–	Methotrexate	21	16
Autologous	1	8				Mycophenolate	50	68

ALL, acute lymphoblastic leukemia; AML, acute myeloblastic leukemia; ATG, antithymocyte globulin; CGD, chronic granulomatous disease; GvHD, graft‐vs.‐host disease; HLH, hemophagocytic lymphohistiocytosis; SCT, stem cell transplantation; M, model‐building dataset (*n* = 319), used for model building and covariate analysis; SCID, severe combined immunodeficiency syndrome; V, validation dataset (n = 75), used for assessing the predictive ability of the model.

Positive for cytomegalovirus, Epstein‐Barr virus, or adenovirus was defined as detectable virus post‐transplant.

### The type of conditioning affects the reconstitution

**Figure 3:**
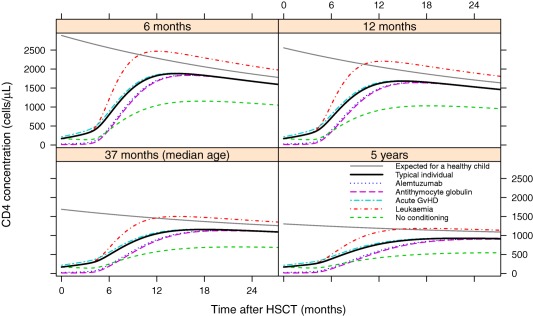
The effects of the significant covariates (*P* < 0.005, based on a likelihood ratio test) on the CD4 reconstitution of patients of 6 months, 12 months, 37 months (median age), and 5‐years‐old at the time of hematopoietic stem cell transplantation (HSCT). A typical individual is one who is not in each of the covariate groups listed. The expected curve of a healthy child uses the function for *N*(τ) given in the Methods section.^2^ Each other trajectory gives the effects of the significant covariates, included through the stepwise covariate model procedure. Conditioning drugs alemtuzumab (*n* = 158) and antithymocyte globulin (*n* = 10), and acute graft‐vs.‐host disease (*n* = 102) affect initial number of cells, whereas leukemia (*n* = 95) and having no conditioning (*n* = 41) affect long‐term reconstitution.

Parameter estimates for T‐cell concentration at the time of HSCT were lower with two of the conditioning drugs, alemtuzumab and antithymocyte globulin (ATG). For patients having neither of these drugs (*n* = 151), the model parameter estimate for the mean initial CD4 concentration was 178 cells/μL, whereas for those who had alemtuzumab (*n* = 158), the estimated mean was decreased by 83% to 30.6 cells/μL (*P* < 0.001), and for those who had ATG (*n* = 10), it was decreased by 95% to 8.4 cells/μL (*P* < 0.001). This resulted in a delayed reconstitution (**Figure** 3).

Although the estimated initial mean number of cells in patients who received no conditioning (*n* = 41) was unaffected, reconstitution was found to result in a lower long‐term concentration, below that expected of a healthy child (**Figure** 3).

### Patients with leukemia have higher CD4 concentrations

Patients with leukemia (*n* = 95) were estimated to have a higher long‐term CD4 concentration after HSCT than those with other conditions (*P* < 0.001; **Figure** 3). Both patients with lymphoblastic leukemia (*n* = 45) and patients with myeloid leukemia (*n* = 50) were found to have significantly higher long‐term CD4 concentrations, although there was no significant difference between them (*P* = 0.23).

### Having acute graft‐vs.‐host disease is associated with a higher initial CD4 concentration

**Figure 4:**
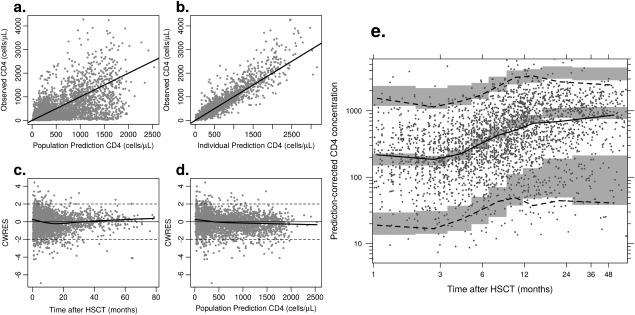
Diagnostic plots for the model. (**a** and **b**) give population and individual predictions vs. observations; (**c** and **d**) give conditional weighted residuals (CWRES) against time and population prediction, respectively; (**e**) gives a visual predictive check: dots give the observed data, the solid black line the observed median, and the dashed black lines the observed 95% prediction intervals. The gray shaded areas give the 95% confidence intervals for the predicted median and for the predicted 95% prediction intervals.

The estimated mean initial CD4 concentration for patients who had acute graft‐vs.‐host disease (GvHD; *n* = 102) was 28% higher than those for whom there was no reported GvHD (*P* < 0.001; **Figure** 3). Model diagnostics from the full covariate model are given in **Figure** 4.

### Bayesian predictions of reconstitution trajectories

A separate validation dataset that had not been used for model building was used to assess the predictive ability of the model. To illustrate the model's potential usefulness, individual parameter estimates were generated using CD4 concentrations measured in the 6 months post‐HSCT and the individual's relevant covariates. These individual parameter estimates were used to produce a predicted reconstitution trajectory, which was compared with actual post 6‐month measurements not used in the model for up to 3 years post‐HSCT.

**Figure 5:**
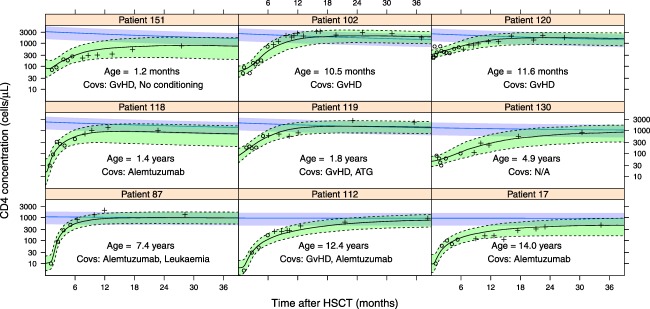
Examples of predicted reconstitution (9 patients of the 75 that were modeled) in which the model achieved a good prediction, listed in age order. The circles are the data points that were used to make the predictions, and the crosses are the data not used in forming predictions for comparison to the predictions. The line is the median prediction, with the green shaded area giving the 90% confidence intervals. The blue line and shaded area are the median and 90% confidence intervals of the expected CD4 concentration of a healthy child of this age. GvHD, graft‐vs.‐host disease; HSCT, hematopoietic stem cell transplantation.

In 81% of the patients (*n* = 61), the model gave a good prediction, with over 75% of the observed data within the model confidence intervals, and the correct trend of CD4 reconstitution identified. Examples of good predictions in nine patients are highlighted in **Figure** 5), whereas predictions for all patients are in **Supplementary Figure S3** online. The highlighted patients were chosen to have ages from across the spectrum of the data and a spread of the covariates used in the model. Patients 102 and 120 were chosen to demonstrate that two individuals with similar age and the same covariates could have substantially different reconstitution pathways predicted by the model, which was guided by early CD4 concentrations. Also of note is patient 130, who from early measurements could be thought to be at risk of poor recovery, but the model showed normal expected long‐term recovery, as confirmed by later observations.

## DISCUSSION

A novel mechanistic model of CD4 T‐cell reconstitution after HSCT in children has been developed. HSCTs are performed in heterogeneous groups of patients each requiring a stratified approach to conditioning and follow‐up treatment. Prolonged CD4 T‐cell count after HSCT leaves patients at risk of mortality and morbidity due to opportunistic infection, so understanding factors associated with reconstitution is vital. Our model now has the potential to be used in a clinical trial setting^11^ or in multivariable analysis of cohort studies^1^ to tease out studied treatment effects from other important covariates. Furthermore, the model has the ability to predict reconstitution on an individual basis. A major part of the clinical management of patients post‐transplant is in immunosuppressant dosing to limit GvHD, whereas preventing graft failure, carried out alongside managing infective and other complications with other (potentially interacting) drug and cell therapies. Monitoring CD4 counts forms an important guide to this process, and Bayesian predictions from our model reported alongside CD4 counts will aid clinicians in making treatment decisions in the post‐transplant period.

Predictions were made from the model for individual patients in another dataset, using only their covariates and data up to 6 months post‐HSCT. Predictions were then validated for up to 3 years after the HSCT, with accurate predictions in 81% of patients tested. Using the individual patient's variance‐covariance matrix, we were able to provide confidence intervals on the predicted trajectory, the size of which being a reflection of the amount of information available on that patient. Predictions were formed using data from the first 6 months. Earlier predictions could be made, but the accuracy of the resulting predictions decreased as the confidence intervals of the predictions increased. Similarly, as new measurements of CD4 concentrations were taken, the predictions could be updated; with each additional data point, the parameter estimates improve and the confidence intervals decrease. With the validating dataset in this study, using data from the first 3 months rather than 6 months increased the size of the confidence intervals by a mean of 10%, whereas using data from the first 12 months decreased confidence intervals by 12%.

Previous studies have reported a median CD4 count in the range of 100–150 cells/μL at 3 months post‐HSCT^3^, ^4^, ^5^, ^22^ and 500–1000 cells/μL at 1 year post‐HSCT,^3^, ^5^, ^9^ which agrees well with the our model output for a typical child aged 37 months of 105 cells/μL at 3 months and 984 cells/μL at 1 year. Similarly, the time taken to reach 500 cells/μL was around 10.1 months (range, 1.1–55.3 months) in a previous study^7^; whereas our model predicts 7.5 months for a median‐aged child varying from 5.3 months for a 1‐year‐old to 14.3 months for a 10‐year‐old child. Hence, the model‐based approach we present builds on previous work by simultaneously analyzing the rate and extent of reconstitution. Furthermore, the model seems to give realistic estimates of mean CD4 T‐cell lifespan of 130, 300, and 550 days for a 1, 10, and 18‐year‐old, respectively. These agree with recent analyses of labeling studies with estimates between 222 and 611 days (range, 167–1245 days).^21^, ^23^

In searching for significant covariates associated with recovery, we first sought to delineate the effect of age, which is a potential confounder because normal CD4 count changes radically with age, and children of different ages will receive HSCTs for different reasons. Rather than correcting each data point for an age‐expected value, as has previously been done,^24^ we chose to scale the model parameters to age‐expected values using biological prior information on thymic output and markers for competition and loss.

Thymic output for age was predicted from a previous study.^13^ The absolute value of the prediction was uncertain due to a constant for Ki67 expression duration, and more recent work has indicated that thymic output could be as little as 10% of that predicted.^21^ As such, we used a scaling factor, thereby retaining the shape of the expected thymic output with age while allowing its magnitude to be informed by the data. Our estimate of 22%, which was previously predicted,^13^ agrees with these later analyses.^21^ In addition, parameters describing thymic recovery post‐HSCT were added, and the model predicting 90% production for age taking around 7 months, matched well the experimental evidence of recovery between 5 and 10 months from both TREC analysis^19^, ^20^ and measures of recent thymic emigrants using CD31 expression.^3^

The covariates listed in **Table** 2 were tested. Alemtuzumab and ATG are given as pretransplantation conditioning to deplete circulating lymphocytes and have long terminal half‐lives (15–21 days for alemtuzumab^25^ and 29.8 days for ATG.^26^) The finding that these drugs were associated with reduced initial CD4 counts was, therefore, unsurprising. Alemtuzumab and ATG decreased the initial number of cells by 84% and 94%, respectively, which is in line with other studies, in which alemtuzumab and ATG were associated with later and slower reconstitution in both children and adults.^22^, ^26^, ^27^ A previous study also found that alemtuzumab caused a significantly longer delay to reconstitution than ATG,^28^ which was not observed in the analysis described here, perhaps because our data included very few patients who received ATG.

Those patients who had no pretransplantation conditioning had a reduced mean long‐term CD4 concentration, which differs from studies showing that reduced conditioning was associated with increased CD4 concentrations.^22^, ^26^, ^27^, ^29^ One possible explanation is that pretransplantation conditioning creates T‐cell space allowing donor T cells to expand more efficiently. Similarly, the finding of increased long‐term CD4 concentration for patients with leukemia could be due to these patients receiving full myeloablative conditioning, leaving more space for donor T‐cell expansion.

Our model also predicted that a raised initial CD4 concentration was associated with incidence of acute GvHD. This agrees with previous studies that found T‐cell depleted grafts to be associated with decreased incidence of acute GvHD.^30^, ^31^, ^32^, ^33^ The association was significant in addition to the changes in the initial mean CD4 concentration caused by alemtuzumab, which was received by 53% of the patients with acute GvHD.

In the covariate analysis, cord blood transplantation (CBT) was not found to be a significant covariate, in agreement with Fernandes *et al*.^34^ In the observed data, patients who have had a CBT (*n* = 48), as opposed to peripheral blood or bone marrow transplants, had a faster reconstitution in the months after the transplant. In the covariate analysis, however, these differences were explained by a combination of the effects of age and pretransplantation conditioning. Patients who underwent CBT were younger, with 60% of the patients under 2‐years‐old (*n* = 29) at the time of HSCT in comparison to 37% of the rest of the model‐building dataset and with a median age at HSCT of 1.5 years in comparison to 3.6 years. They were also less likely to have had alemtuzumab or ATG, with 83% (*n* = 40) having neither, in comparison to 41% of the rest of the transplants. This agrees with other studies, which have found that age^7^ and the omission of ATG^35^ can explain the differences observed in the reconstitution of patients after CBT.

In conclusion, a mechanistic model was developed that predicted on an individual basis the long‐term immune reconstitution of CD4 T cells after HSCT. For the first time, the model brought together many aspects of the immune system after an HSCT, including homeostatic mechanisms, changes to thymic output, loss and proliferation with age, and impaired thymic production of T cells in the months after HSCT. By using this biological prior knowledge in the model, parameter estimates were able to delineate expected age effects from disease and treatment‐specific covariates, in addition to separating CD4 production from loss. These predictions allow for a more informed assessment of the potential long‐term position of the patient and could be used to inform clinicians of the necessity of a change in regimen for that particular patient.

To our knowledge, this was the first time a mechanistic model has been used to predict long‐term reconstitution after HSCT in children. As we enter an era of electronic hospital records, there is the potential to use these data directly to provide predicted reconstitution trajectories automatically for children after HSCT, creating a useful tool to inform on the clinical management of these patients.

## METHODS

### Data

The dataset used for model building and covariate analysis was collected during routine clinical practice between 2005 and 2011 by the Blood and Marrow Transplant Unit at the Great Ormond Street Hospital for Children NHS Trust. The validation dataset was collected in the same manner between 2010 and 2014. The study was approved by the Great Ormond Street Hospital Institutional Review Board and the parents of the patients provided written informed consent for their data to be used in the database according to the Declaration of Helsinki. The data comprised CD4 T‐cell concentrations taken at regular intervals for up to 7 years after HSCT.

In the modeling dataset, there were 288 patients who had 319 transplants among them. There were 2,928 CD4 concentrations in total with a median of 8 samples (range, 1–43 samples) taken post‐transplantation. In this dataset, 24% of the patients died within the 1–6 year follow‐up period; of which 36% died from infection, 35% from disease relapse, and 15% from acute GvHD. In the validation dataset, there were 75 patients. A breakdown of the demographics of both datasets is given in **Table** 2.

### Model building

The rate of change in *X*(*t*) (the CD4 T‐cell concentration with time *t* after the HSCT) is:

(1) $$\frac{d}{dt}X=\lambda -dX+pX$$

where λ zero‐order thymic output of T cells; *d* first‐order cell loss rate; and *p* first‐order proliferation rate. Biological prior knowledge was then incorporated into the model.

### Homeostatic mechanisms and age affect proliferation and loss

T‐cell populations are maintained through proliferation and loss. To survive and proliferate, CD4 T cells require interactions with resources, such as cytokines^14^, ^36^ and self‐peptide major histocompatibility complex class II complexes.^37^ Homeostasis is then maintained through competition for these resources^15^, ^16^ with proliferation and loss of concentration dependence. To model this, we simplify a previous model for the competition effects in T‐cell homeostasis.^17^ The resulting exponential dependence on concentration represents the simplest non‐negative functions, whereas adding the fewest parameters to the model. Furthermore, the rate of turnover of T cells decreases with age^13^, ^18^; we use the decrease in Ki67 with age as a marker for proliferation to inform the timescales for these changes,^12^ giving:

(2) $$\begin{array}{l} p=p_{0}y\left(\tau \right)e^{c_{p}\left(1-\frac{X\left(t\right)}{N\left(\tau \right)}\right)}\\ d=d_{0}y\left(\tau \right)e^{c_{d}\left(\frac{X\left(t\right)}{N\left(\tau \right)}-1\right)}, \end{array}$$

where $N\left(\tau \right)=924+2354e^{\left(-0.001012\tau \right)}$ is the expected total CD4 concentration in cells/μL for a healthy child of age τ days,^2^ and $y\left(\tau \right)=0.02e^{\left(-0.00027\tau \right)}$ is the proportion of CD4 cells expressing Ki67 with age.^13^

### Thymic output changes with age

The thymus reaches full size at 1 year, after which thymic output decreases rapidly as thymic epithelial space involutes by 70% over the next 20 years.^13^, ^38^ This change in production was characterized mathematically^12^ using TREC dynamics and removing dilution from proliferation, leaving the following function describing the thymic output as a function of age τ:

(3) $$\lambda_{age}\left(\tau \right)=\frac{y\left(\tau \right)N_{n}\left(\tau \right)\gamma }{0.02\eta \left(c-\gamma \right)},$$

where $N_{n}\left(\tau \right)=496.5+2074e^{\left(-0.000869\tau \right)}$ is the expected naïve CD4 concentration in cells/μL for a healthy child of age τ days,^2^ η = 0.52 days is the duration of Ki67 expression, and *c* = 0.25 and γ = 0.08 give the average TREC content of naïve T cells as they leave the thymus and of the naïve T‐cell pool, respectively.

### Thymic output is altered by the hematopoietic stem cell transplantation

Evidence suggests that, after HSCT, thymic output of T cells takes between 6 and 10 months to recover.^3^, ^19^, ^20^, ^39^ We used a sigmoidal function to model this:

(4) $$\begin{array}{c} \Delta_{HSCT}\left(t\right)=\frac{1-exp\left[\frac{-2t}{\lambda_{h}}\right]}{1+exp\left[\lambda_{r}(1-t/\lambda_{h}\right]} \end{array}$$

where $\lambda_{h}$ gives the time after HSCT that thymic output increases, and $\lambda_{r}$ gives the rate of this increase. A sigmoidal function of this form was used because it was found during model development that models in which thymic output could be zero immediately post‐transplant fitted the data much better. The rate parameter for a standard logistic function was, therefore, increased in order to make the curves much steeper so that thymic output immediately post‐HSCT would be zero or close to zero. This made model fitting unstable and gave unrealistic estimates for the recovery rate of thymic output post‐HSCT, based on evidence from TREC analysis. Similarly, this function fitted the data better than a Hill function. **Supplementary Figure S2** online demonstrates the effects of the parameters $\lambda_{h}$ and $\lambda_{r}$.

### The complete model

The complete model is then:

(5) $$\frac{d}{dt}X\left(t,\tau \right)=\lambda \left(t,\tau \right)-d\left(X,t,\tau \right)X\left(t,\tau \right)+p\left(X,t,\tau \right)X\left(t,\tau \right),$$

where

(6) $$\lambda \left(t,\tau \right)=\lambda_{0}\lambda_{age}\Delta_{HSCT}\left(t\right)$$

(7) $$p\left(X,t,\tau \right)=y\left(\tau \right)p_{0}e^{c_{p}\left(1-\frac{X\left(t\right)}{N\left(\tau \right)}\right)}$$

(8) $$d\left(X,t,\tau \right)=y\left(\tau \right)d_{0}e^{c_{d}\left(\frac{X\left(t\right)}{N\left(\tau \right)}-1\right)}$$

with *X*(0,τ) the estimated parameter *X_0_*.

### Model fitting

Identifiability analysis using the FME package^40^ in R version 2.15.1,^41^ demonstrated that the effects of the parameters for the strength of competition for resources, c_p_ and c_d_, on the curve of the reconstitution could be absorbed into other parameters. As such, they were fixed to one. All other parameters were estimated with both fixed and random effects, with a full variance‐covariance matrix estimated for the random effects. All model parameters were lognormally distributed and the additive residual error model was applied to log‐transformed CD4 counts.

Nonlinear mixed effects modeling with NONMEM version 7.3^42^ was used. The Importance Sampling expectation‐maximization algorithm^43^ was used. Quality of fit was assessed using diagnostic plots (**Figure** 4). Conditional weighted residuals were approximately normally distributed with mean 0 and variance 1 and independent of time and population prediction. Model misspecification was assessed with a visual predictive check, whereby for each data point in the observed data, 600 data points were simulated from the model using the parameter estimates and variance‐covariance matrix. Having ascertained that the model fit the observed data, this ascertained that model‐simulated data matched the observed data albeit with some deviations at later time points. The visual predictive check was produced using PsN version 3.5.3.^44^

### Covariate model building

A total of 34 covariates were chosen that could potentially influence CD4 reconstitution. These fell into the following categories: diagnosis, pretransplantation conditioning, stem cell source, post‐transplant immunosuppressant regimen, and post‐transplant outcomes (e.g., GvHD, graft failure, and presence of viral infection). All covariates were dichotomous and entered the model as follows: $typical\;parameter\;value\;\times \left(1+\;\theta_{cov}\right)$, where $\theta_{cov}$ was the proportional change in typical parameter value in the presence of the covariate. Further details are given in the Supplementary Materials online. These were included using stepwise covariate model building,^46^ whereby, during the forward search, covariates were tested on each parameter, with the one yielding the best fit retained for the next step until no more covariates led to significant improvement in fit. During backward elimination, covariates from the forward search are excluded in a stepwise manner with stricter significance criteria. Because models are nested, a likelihood ratio test at each step with the difference in −2ln (likelihood) asymptotically $\chi_{n}^{2}$ distributed (where *n* is the difference in the number of nested parameters). In the forward search, we used an inclusion criterion of *P* < 0.01 and in the backward elimination *P* < 0.005, and stepwise covariate model was implemented using PsN version 3.5.3.^44^

### Predicting reconstitution from early data and individual covariates

The population parameter means and variances found from the initial model fitting were used as the priors. The posterior individual‐level parameter values using data from the first 6 months post‐transplant were then found through expectation‐only importance sampling steps using the covariate model and parameter estimates from the model‐building dataset. In this process, the conditional (posterior) mean and variance of individual parameters were evaluated by Monte Carlo sampling, and the likelihood of these individual parameters was maximized given the fixed population means and variances and the individual's observed data.^43^

Predicted trajectories were then formed from these individual parameters: 500 sample parameter sets were simulated from the parameter means and their variance‐covariance matrix. From these sample curves, the median and confidence intervals were found for the trajectory of that individual's CD4 T‐cell reconstitution.

### Code availability

An R script is included in the Supplementary Materials online that produces predictions for an individual child. It formats data, runs the NONMEM script with the model, also in the Supplementary Materials online, and finally produces a graphical output of the prediction for that patient.

## SOURCE OF FUNDING

R.H. was supported by an Engineering and Physical Sciences Research Council Life Sciences Interface Doctoral Training Centre studentship at the Centre for Mathematics and Physics in the Life Sciences and Experimental Biology (CoMPLEX). J.S. was supported by a United Kingdom Medical Research Council Fellowship (grant G1002305). The research was supported at institution level *by the National Institute for Health Research Biomedical Research Centre at Great Ormond Street Hospital for Children NHS Foundation Trust and University College London*.

## CONFLICT OF INTEREST

The authors declared no conflict of interest.

## AUTHOR CONTRIBUTIONS

J.F.S., R.L.H., P.V., N.K., and R.C. wrote the manuscript. J.F.S., R.L.H., and R.C. designed the research. J.F.S., R.L.H., P.V., and R.C. performed the research. J.F.S., R.L.H., N.K., and R.C. analyzed the data.

## Supporting information

Supporting Information
