# Supplementary material for: Predicting CD4 T‐Cell Reconstitution Following Pediatric Hematopoietic Stem Cell Transplantation
Source: Clin Pharmacol Ther. 2017 May 26;102(2):349–57. doi: 10.1002/cpt.621 (PMC5579758; doi:10.1002/cpt.621)
Supplement: Supplementary file 1 — Supporting Information [file CPT-102-349-s001.docx]

## 1 Supplementary material

##### Further details on the covariate model building

##### The following covariates were treated as categorical variables during stepwise covariate model building:

1. Demographics: Sex, transpant number (e.g. was this the first or second transplant?), diagnosis

2. Pre-transplant conditioning: alemtuzumab, ATG, busulfan, treosulfan, melphalan, fludaabine, cyclofosfamide, total body irradiation, no conditioning

3. Donor type and source

4. Post-transplant immunosuppressants: cyclosporin, methotrexate, mycophenolate, prednisolone, tacrolimus

5. Post-transplant outcomes: GvDH, chimerism, graft failure, presence of viral infection (cytomegalovirus, Epstein Barr virus)

The following covariates that were deemed potentially interesting yet did not turn out to be significant when categorised were further checked as presence/absence: GvHD (rather than grade), cord blood separated from any other stem cell source. Neither covariate was significant following this procedure.

##### A note on age scaling of Ki67

##### CD4 cell loss and proliferation were modelled as age dependent, using a function that had been derived for the proportion of T cells expressing Ki67 with age. Another explanation could be allometric scaling, whereby the total metabolic rate of an organism scales with weight and total lymphocyte number scales with *weight*/ln(*weight***c*) where *c* is a constant with units 1/weight and therefore rates of lymphocyte dynamics scale with weight. The two models (age dependence and allometric scaling) produce very similar results for the decline in proliferation and loss (see Supplementary Fig. 1). To use allometric scaling however requires body weight to be known at each measurement, which was not available in these data. We tested an assumed weight for age relationship and found equivalent fits (-2 log likelihood of 404.5 and 402.0 for the age and weight models respectively) and therefore decided to use the model for the decline with age that had been used previously.

##### Parameterisation of the post-transplant recovery in thymic output

##### Supplementary Fig. 2 shows the estimated shape of the thymic output recovery curve, and associated effect of changing parameters.

##### Performance of the model for Bayesian predictions

##### In Supplementary Fig. 3 predictions for all subjects in the prediction validation cohort with sufficient data set are given. We identified 6 patients for whom the predictive performance was poor, these were patients: 24, 38, 69, 85, 101 and 161. Whilst for patients 24 and 101 the model under-estimated reconstitution, with patients 38, 69, 85 and 161, the observed trajectories fell below the predicted interval. Patient 38 was a 6 year old with the only significant covariate being ATG conditioning, patient 69 was a 1 year old with no significant covariates, patient 85 was a 3 year old with GvHD and alemtuzumab conditioning as significant covariates, and patient 161 had covariates of GvHD and ATG conditioning. At present there is no clear link between poor preditions and patient factors, but as this cohort ages and hence larger numbers can be included, future work will entail detailed investigation into those patients with poor predictions.

## 2 R code

library(lattice)

library(FME)

library(latticeExtra)

rm(list=ls())

#######################################################################

##### Input Patient Characteristics ###################################

# Patient Hostpital Number or another numeric identifier

Patient_ID=1004

# The Age (in days) on the day of the HSCT

Age_at_HSCT = 4676

## Input diagnosis:

# 1: Leukaemia (any form)

# 2. Any other diagnosis

Diagnosis = 2

## Conditioning details:

# 1: Alemtuzumab

# 2. ATG

# 3. any other form of conditioning (e.g. TBI, ACD45, other drugs etc)

# 4. No conditioning at all

Conditioning = 1

## Acute GvHD Status

# 1: Never had acute GvHD

# 2: Had acute GvHD

Acute_GvHD_status = 1

#######################################################################

##### Input CD4 concentration data ####################################

# Formats:

# Times in days following HSCT (with day 0 as the day of HSCT)

# Concentrations in cells/uL (i.e. should be roughly in the range of 10 to 4000)

# Patient weights at each measurement if using allometric scaling model. Else a list of zeros of the same length as the Times and Concentrations.

Times = c(20,176,204,239,281,323)

Concentrations = c(10,120,160,340,680,1010)

Weights = c(0,0,0,0,0,0)

#######################################################################

##### Simulation of data characteristics ##############################

# The number of sample parameter values formed:

# - More samples gives a more accurate representation

# - Fewer samples is faster

# - Somewhere between 300 and 1000 is recommended

samples=500

# The size of the confidence interval of the trajectory to plot (%):

Confidence_Interval = 68

#######################################################################

##### Model File Name #################################################

Model = "CD4_individual_prediction"

#######################################################################

##### Set directory for modelling #####################################

Directory = "~/Documents/R/CD4/Predictions"

# Model file should be saved in this directory in format: runModel.mod

#######################################################################

##### Create directory for individual patient for prediction ##########

Individual_Directory = paste(Directory,"/Patient_",Patient_ID,sep="")

system(paste("mkdir ",Individual_Directory,sep=""))

setwd(Individual_Directory)

#######################################################################

#######################################################################

##### Carry out parameter estimation and simulation ###################

#######################################################################

#######################################################################

##### Create data set given information above #########################

nonmem_data <- data.frame(

ID = Patient_ID,

TIME = c(0,Times),

DV = c(0,log(Concentrations)),

EVID = c(2,rep(0,length(Times))),

AGE = c(0,Times) + Age_at_HSCT,

WT = c(0,Weights),

GVHYN = 0,

COND=0,

ALEM=0,

ATG=0,

LEUK=0)

if (Conditioning == 1){nonmem_data$ALEM <- 1}

if (Conditioning == 2){nonmem_data$ATG <- 1}

if (Conditioning == 4){nonmem_data$COND<-1}

if (Diagnosis == 1){nonmem_data$LEUK <- 1}

if (Acute_GvHD_status == 2){nonmem_data$GVHYN <- 1}

names(nonmem_data)[1] <- "#ID"

write.csv(nonmem_data,"nonmemdata_log_predictions.csv",row.names=FALSE,quote=FALSE)

## ############################################################################ Carry out model fitting in NONMEM ###############################

system(paste("cp ",Directory,"/run",Model,".mod run",Model,".mod",sep=""))

system(paste("execute run",Model,".mod -directory=NM_run",Model,sep=""))

system(paste("rm -r NM_run",Model,sep=""))

#######################################################################

##### Read back in NONMEM results #####################################

phi <- read.table(paste("run",Model,".phi",sep=""),header=T,skip=1)

sigma <- read.table(paste("run",Model,".ext",sep=""),header=T,skip=1)[1,"SIGMA.1.1."]

#######################################################################

##### Create sample parameter sets from NONMEM results ################

nparam=6 # Number of parameters with random effects

varcovar_list <- list()

for (i in 1:nparam){

for (j in 1:nparam){

if (j>i) {name <- paste("PHC",j,i,"",sep=".")}

else {name <- paste("PHC",i,j,"",sep=".")}

varcovar_list <- append(varcovar_list,name)}}

varcovar_list <- unlist(varcovar_list)

mean_list <- list()

for (i in 1:nparam){

mean_name <- paste("PHI",i,"",sep=".")

mean_list=append(mean_list,mean_name)}

mean_list <- unlist(mean_list)

parMean <- unlist(phi[,mean_list])

parCovar <- matrix(data=unlist(phi[,varcovar_list]),nrow=nparam)

sample_params <- data.frame(Norm(parMean=parMean,parCovar=parCovar,num=samples

))

names(sample_params) <- c("lambda","d","p","X0","LH","LR")

real_sample_params <- exp(sample_params)

#######################################################################

##### Simulate data from sample parameter sets ########################

sample_param <- real_sample_params[1,]

pars <- list(lambda = sample_param$l,

d = sample_param$d,

p = sample_param$p,

X0 = sample_param$X0,

LH = sample_param$LH,

LR = sample_param$LR)

#Define model to solve, including time range, differential equations, starting

estimates for compartments

solveCD4 <- function(pars, times=seq(0,3000,by=10)){

derivs <- function(t, state, pars) {

with(as.list(c(state,pars)),{

Age = Age_at_HSCT

V = (497+2070*exp(-0.00087*(t+Age)))

V2 <- (924+2354*exp(-0.001012*(t+Age)))

L = lambda*exp(-0.00027*(t+Age))*V*0.02*(1-exp(-2*t/LH))/(1+exp(LR*(1-t/LH)))*0.02/0.0221

D = d*0.018*exp(-0.00027*(t+Age))*exp(1*(X/V2-1))

P = p*0.02 *exp(-0.00027*(t+Age))*exp(1*(1-X/V2))

dX <- L - X*(D-P)

return(list(c(dX)))

})

}

state <- c(X = pars$X0)

return(ode(y=state,times=times,func=derivs,parms=pars))

}

# Carry out estimation from model

out <- data.frame(solveCD4(real_sample_params[1,]))

for (i in 2:samples){out_new <- data.frame(solveCD4(real_sample_params[i,]))

out[,i+1] <- out_new$X

}

#######################################################################

#######################################################################

##### Plot Results ####################################################

#######################################################################

#######################################################################

##### Calculate confidence intervals for plots ########################

CI.lower= (50 - Confidence_Interval*0.5)/100.0

CI.higher=(50 + Confidence_Interval*0.5)/100.0

out$lower <- apply(out[,2:ncol(out)],1,quantile,probs=c(CI.lower))*exp(-sigma)

out$median <- apply(out[,2:ncol(out)],1,quantile,probs=c(0.5))

out$higher <- apply(out[,2:ncol(out)],1,quantile,probs=c(CI.higher))*exp(sigma

)

out <- as.data.frame(out[,c("time","lower","median","higher")])

write.csv(out,paste("Predicted Trajectories",Patient_ID,".csv",sep=""))

#######################################################################

##### Include CD4 concentration with age ##############################

CD4_expected <- data.frame(

AGE = 1:8000)

CD4_expected$CD4 <- 924+2354*exp(-0.001012*CD4_expected$AGE)

CD4_expected$CI05 <- 0.469*CD4_expected$CD4

CD4_expected$CI95 <- 1.687*CD4_expected$CD4

out$AGE <- Age_at_HSCT + out$time

out <- merge(out,CD4_expected)

out$min <- do.call(pmin,data.frame(out$lower,out$CI05))

out$time <- out$time/30.5

#######################################################################

##### Plot results and CD4 concentration for age and save #############

pdf(file=paste("Predicted trajectory ",Patient_ID,".pdf",sep=""),width=8,

height=5)

xyplot(CD4~time, out,type="l", scales=list(x=list(limit=c(0,39), at=c(6,12,18,24,30,36)), y=list(log=T,limit=c(3,6000), at=c(10,30,100,300,1000,3000))), xlab=list(label="Time after HSCT (months)",

cex=1.5), ylab = list(label=expression(paste("CD4 concentration (cells/",mu

,"L)")),cex=1.5)) +

as.layer(xyplot(CI95~time,out,type="l",lty=2,scales=list(y=list(log=T)), panel=panel.xyarea, border=0,origin=0,aspect=’xy’,col=rgb

(0,0,1,0.2))) +

as.layer(xyplot(CI05~time,out,type="l",lty=2, scales=list(y=list(log=T)), panel=panel.xyarea, border=0,origin=0,aspect=’xy’,col="white",

col.line="white")) +

as.layer(xyplot(higher~time,out,type="l",lty=2, scales=list(y=list(log=

T)), panel=panel.xyarea, border=0,origin=0,aspect=’xy’,col=rgb

(0.1,1,0,0.3))) +

as.layer(xyplot(lower~time,out,type="l",lty=2, scales=list(y=list(log=T)), panel=panel.xyarea, border=0,origin=0,aspect=’xy’,col="white"))+

as.layer(xyplot(lower~time,out,type="l",lty=2, scales=list(y=list(log=T

)), panel=panel.xyarea, border=0,origin=0,aspect=’xy’,col=rgb

(0,0,1,0.2))) +

as.layer(xyplot(min~time,out, scales=list(y=list(log=T)), panel=panel.

xyarea,border=0, origin=0,aspect=’xy’,col="white", col.line="white"))+

as.layer(xyplot(higher~time,out,type="l",lty=2, col="black", scales=

list(y=list(log=T)))) +

as.layer(xyplot(lower~time,out,type="l",lty=2,col="black", scales=list(y=list(log=T)))) +

as.layer(xyplot(median~time,out,type="l",lty=1,col="black", scales=list(y=list(log=T)))) +

as.layer(xyplot(exp(DV)~TIME/30.5,nonmem_data,cex=0.7, type="p", col="

black", scales=list(y=list(log=T))))

dev.off()

#######################################################################

#######################################################################

## 3 NONMEM code

$PROBLEM Paediatric HSCT CD4 T cell Reconstitution Individual Predictions

;____ Data Input and setup of problem _______________________________________

$INPUT ID TIME DV EVID AGE WT GVHYN COND ALEM ATG LEUK

$DATA nonmemdata_log_predictions.csv IGNORE=@

$SUBROUTINE ADVAN13 TOL=9

$MODEL COMP=(CENTRAL) COMP=(VAGE) ; Second compartment gives time varying

age

$PK

;____ Initiate population level parameters for the model ____________________

TVL = THETA(1)

TVD = THETA(2)

TVPR = THETA(3)

TVINT = THETA(4)

TVLH = THETA(5)

TVLR = THETA(6)

;____ Modelling covariate effects on population level parameters ____________

IF(ALEM.EQ.0) INTALEM = 1

IF(ALEM.EQ.1) INTALEM = (1 + THETA(7))

IF(ATG.EQ.0) INTATG = 1

IF(ATG.EQ.1) INTATG = (1 + THETA(8))

IF(GVHYN.EQ.0) INTGVHYN = 1

IF(GVHYN.EQ.1) INTGVHYN = (1 + THETA(9))

IF(LEUK.EQ.0) LLEUK = 1

IF(LEUK.EQ.1) LLEUK = (1 + THETA(10))

IF(COND.EQ.0) PRCOND = 1

IF(COND.EQ.1) PRCOND = (1 + THETA(11))

TVINT = TVINT * INTALEM * INTATG * INTGVHYN

TVL = TVL * LLEUK

TVPR = TVPR * PRCOND

;____ Mu modelling to improve model run-times _______________________________

MU_1 = LOG(TVL)

MU_2 = LOG(TVD)

MU_3 = LOG(TVPR)

MU_4 = LOG(TVINT)

MU_5 = LOG(TVLH)

MU_6 = LOG(TVLR)

;____ Including Age effects and individual random effects on parameters ____

;________ Using Bains age only model

LAMBDA0 = EXP(MU_1 + ETA(1))

DELTA0 = EXP(MU_2 + ETA(2))

PROL0 = EXP(MU_3 + ETA(3))

;________

INT = EXP(MU_4 + ETA(4))

LH = EXP(MU_5 + ETA(5))

LR = EXP(MU_6 + ETA(6))

CD = 1

CP = 1

A_0(1) = INT

;______ Time varying age definition _________

IF(NEWIND.NE.2) OTIM=0

IF(NEWIND.NE.2) OAGE=0

SL = 0

IF(TIME.GT.OTIM) SL= (AGE-OAGE)/(TIME-OTIM)

A_0(2) = AGE

;______ Define the differential equation for the model _________

$DES

;______ Age extrapolation between observations ________

DADT(2) = SL

VAGE = A(2)

;_____ Naive (V) and total (VT) CD4 T cell concentration with age __

VT = 924 + 2354*EXP(-0.001012*VAGE)

V = 496.5 + 2074*EXP(-0.000869*VAGE)

;_____ Update age varying parameters

L = LAMBDA0* 0.02*EXP(-0.00027*VAGE) * V / 0.0221 * 0.02

D = DELTA0* 0.02*EXP(-0.00027*VAGE)

PR = PROL0* 0.02*EXP(-0.00027*VAGE)

;______ Differential eq of CD4 turnover ________________

DADT(1) = L * (1-EXP(-2*T/LH))/(1+EXP(LR*(1-T/LH))) - A(1)*(D*EXP(CD*(A(1)/VT

-1)) - PR * EXP(CP*(-A(1)/VT+1)))

;______ Compare the model output (IPRED) to the observed data (Y)

$ERROR

OAGE = AGE

OTIM = TIME

;

IPRED = A(1)

IPRED = LOG(IPRED)

Y = IPRED + EPS(1)

;____ Initial estimates for the population level parameters _________________

$THETA

(0,0.215899) ; 1. L,thymic output

(0,0.476551) ; 2. D,loss

(0,0.206862) ; 3. PR,proliferation

(0,168.269) ; 4. A_0,Initial concentration of cells

(0,132.796) ; 5. LH,time to recovery in thymic output

(0,9.66033) ; 6. LR,rate of recovery of thymic output

(-1,-0.84227) ; 7. INTALEM,effects of alemtuzumab on A_0

(-1,-0.939104) ; 8. INTATG,effects of ATG on A_0

(-1,0.283285) ; 9. INTGVHYN,effects of acute GvHD on A_0

(-1,1.31896) ; 10. LLEUK,effects of leukaemia on thymic output

(-1,-0.897173); 11. PRCOND,effects of no conditioning on proliferation

;_____Initial estimates for the random effects var-covar matrix

$OMEGA BLOCK(6)

1.56783

0.530595 1.61526

0.219636 0.355109 0.25062

0.401924 0.340372 0.152781 1.31804

0.576996 -0.442017 -0.0900883 0.816359 1.27365

0.154069 0.42937 -0.0574654 -0.882793 -0.757363 1.22194

;______Initial estimates for the random effects var-covar matrix

$SIGMA 0.218871

;____ Estimation algorithm to be used (EONLY=1 implies Expectation Only) ____

$ESTIMATION METHOD=IMP INTERACTION EONLY=1 ISAMPLE=10000 IACCEPT=0.1 NITER=10

PRINT=1

;____ Output results of the model-fitting into a table ______________________

$TABLE ID TIME DV PRED IPRED CWRES EVID ETA1 ETA2 ETA3 ETA4 ETA5 ETA6 OBJI NOPRINT NOAPPEND ONEHEADER FILE=sdtab_individual_predictions**List of Supplementary Figures**

Supplementary Figure 1: Comparison between Bains model for proliferation decline with age and allometric scaling model with weight for age when applied to previous data for the proportion of CD4 T cells expression Ki67 expression across childhood.

Supplementary Figure 2: Effects of the parameters λ*r* and λ*h* on the sigmoidal function for the

delay in thymic output in the months following HSCT. In each panel, the function is plotted

with the parameter estimates found through fitting to data (λ*r* = 9*.*7, λ*h* = 133) and with individual parameter either increased or decreased by 25%.

Supplementary Figure 3: Predicted reconstitution trajectories and expected CD4 concentrations for age for all patients in the validation dataset for which predictions were made, ordered by age at time of HSCT.
